# Enzymatic characterization of two acetyl-CoA synthetase genes from *Populus trichocarpa*

**DOI:** 10.1186/s40064-016-2532-7

**Published:** 2016-06-21

**Authors:** Shan Cao, Hui Li, Xiaoyun Yao, Lihong Li, Luyao Jiang, Qiang Zhang, Jiaxue Zhang, Di Liu, Hai Lu

**Affiliations:** College of Life Sciences and Biotechnology, Beijing Forestry University, No. 35 Qinghua East Road, Haidian District, Beijing, 100083 People’s Republic of China; National Engineering Laboratory for Tree Breeding, Beijing, 100083 People’s Republic of China

**Keywords:** Acetyl-CoA synthetase (ACS), Prokaryotic expression, Enzyme activity, *Populus trichocarpa*

## Abstract

The acetyl-CoA synthetase (ACS) family is a subfamily of adenylate-forming enzymes, which has a close evolutionary relationship with the 4-coumarate:CoA ligase (4CL) family. In this study, two ACS genes were cloned from *Populus trichocarpa* and were named *PtrACS1* and *PtrACS2.* Bioinformatics characterization of *PtrACS1* and *PtrACS2* showed that they contained the key ACS residues and a putative peroxisome targeting sequence 1 (PTS1) at the end of the C-terminal sequence. Real-time PCR results showed that *PtrACS*1 and *PtrACS*2 were expressed in the phloem, xylem, leaves, and roots of one-year-old *P. trichocarpa*, but were expressed primarily in the leaves. The ACS enzyme activity was higher in leaves than other tissues in *P. trichocarpa*. Two overexpressed recombinant proteins showed no catalytic activity toward the substrates of 4CL, but did have notable catalytic activity toward sodium acetate and substrates of ACS. The relative activities of PtrACS1 and PtrACS2 were 194.16 ± 11.23 and 422.25 ± 21.69 μM min^−1^ mg^−1^, respectively. The *K*_m_ and *V*_max_ of *PtrACS*1 were 0.25 mM and 698.85 μM min^−1^ mg^−1^, while those for *PtrACS*2 were 0.72 mM and 245.96 μM min^−1^ mg^−1^, respectively. Our results revealed that both proteins belong to the ACS family, and provide a theoretical foundation for the identification and functional analysis of members of the adenylate-forming enzyme superfamily.

**Electronic supplementary material:**

The online version of this article (doi:10.1186/s40064-016-2532-7) contains supplementary material, which is available to authorized users.

## Background

The adenylate-forming enzyme superfamily is characterized by the presence of a highly conserved putative AMP-binding domain (PROSITE PS00455) and a shared ATP-dependent, two-step reaction mechanism as follows (Schneider et al. 2005): Acid + ATP → Acyl-AMP + PPi (REACTION 1) and Acyl-AMP + CoA → acyl-CoA + AMP (REACTION 2), which contributes to the biosynthesis or degradation of diverse compounds such as fatty acids, amino acids, and a variety of secondary metabolites. In fact, diverse proteins such as fatty acyl-CoA synthetases, acetyl-CoA synthetases (ACS), 4-coumarate:CoA ligases (4CL), chlorobenzoate:CoA ligase, non-ribosomal polypeptide synthetases, and firefly luciferases are classified in one superfamily of adenylate-forming enzymes (Stuible and Kombrink 2001).

4CL is an important enzyme in lignin biosynthesis. It plays a key role in general phenylpropanoid metabolism, which is the link between lignin precursors and branched products. 4CL catalyzes the activation of various cinnamic acid derivatives (coumarate, caffeate, and ferulate) to form their corresponding CoA esters, and these activated phenolic acids serve as precursors for the biosynthesis of lignin (Stuible and Kombrink 2001; Weisshaar and Jenkins 1998). Previous studies showed that 4CL enzymes are encoded by multigene families in all vascular plants (Hamberger et al. 2007; Lindermayr et al. 2002; Kumar and Ellis 2003), and that isoenzymes of 4CL had differential enzymatic activity toward different hydroxycinnamyl substrates (Stuible and Kombrink 2001).

Sequence analysis showed that 4CL isoenzymes share structural similarities to ACS, such as a conserved substrate binding domain and Box I and II domains (Ehlting et al. 2001). However, determination of the classification of a gene family through sequence alignment analysis alone is not sufficient. In the *Arabidopsis* and *Populus* model plants, a number of genes encoding adenylate-forming enzymes are annotated as being closely related to 4CL despite having unknown specific biochemical functions. Most of these 4CL-like enzymes contain peroxisome targeting sequence 1 (PTS1) sequences in the C-terminal region, and are therefore predicted to be targeted to the peroxisome (Schneider et al. 2005; Koo et al. 2006). Some 4CL-like genes are not associated with flavonoid biosynthesis or lignification (Raes et al. 2003), and they may not have 4CL activity. In *Arabidopsis*, seven 4CL-like recombinant proteins (At5g63380, At4g05160, At4g19010, At3g48990, At1g20510, At1g62940, and At5g38120) had no measurable catalytic activity toward 4CL substrates, while had catalytic activity to acyl:CoA synthetases (Shockey et al. 2003). Since the *Populus trichocarpa* genome sequence was published, more 4CL-like genes have been found and identified, but most do not have the enzymatic activity of 4CL (Zhang et al. 2015). Which family these 4CL-like proteins belong to and what reactions they catalyze should be explored.

In a previous study of *P. trichocarpa*, 18 4CL-like genes were cloned, five of which were classified into the 4CL family, while the rest were unknown (Shi et al. 2010). In this study, two different genes belonging to the 18 4CL-like genes were cloned from *P. trichocarpa*. Sequence and enzyme characterization analyses revealed that both belonged to the ACS family, and displayed high gene expression levels and ACS enzymatic activity in leaves.

## Methods

### Plant material

One-year-old *P. trichocarpa* samples were obtained from our experimental nursery at the Biochemical and Molecular Biology laboratory, Beijing Forestry University.

### Isolation of RNA and cDNA synthesis

Total RNA was isolated from the leaf of *P. trichocarpa* using the TransZol UP Kit (TransGen Biotech Co., Ltd., Beijing, China) according to the manufacturer’s instructions, and cDNA was synthesized after DNase digestion (Promega, Madison, WI, USA) with the RevertAid First Strand cDNA Synthesis Kit (Thermo Scientific, Waltham, MA, USA) according to the manufacturer’s instructions using a (dT)18 primer.

### Cloning and functional analysis of *P. trichocarpa* ACS genes

The opening reading frames of the cDNAs encoding *PtrACS1* and *PtrACS2* were amplified by PCR. The primers used were as follows, for *PtrACS1*: 5′-GGTACCATGGAGAAATCTGGTTATGGTC-3′ and 5′- GTCGACTCATATCTTGGATTTTACTTGC-3′, and for *PtrACS2*: 5′- GGTACCATGGAGAAAATCTGGTTATGGCC-3′ and 5′-CTGCAGTCACATCTTGGATTTCACTTTC-3′. 3 μg RNA was used for cDNA synthesis. Then PCR was performed in a volume of 25 μL containing ~ 2 μL of the first strand cDNA, 0.75U of *Taq* DNA polymerase, 200 μM dNTP, 1.5 mM MgCl_2_ and 10 pmol of each primer. The PCR conditions were optimized and consisted of an initial denaturation of 5 min at 95 °C, followed by 30 cycles of 30 s at 94 °C, 30 s at 55 °C, and 60 s at 72 °C, with a final extension of 10 min at 72 °C. The PCR products were purified and cloned into the PMD18-T vector (Takara, Shiga, Japan), propagated in *Escherichia coli* (*E. coli*) JM109 and the inserts were confirmed by sequencing. Functional characterizations of the ACS gene sequence were obtained from the Uniprot site (http://www.expasy.org/). The primary structure was predicted using the ExPASy ProtParam server (http://expasy.org/cgi-bin/protparam). Physicochemical parameters such as molecular weight, theoretical isoelectric point, number of positively-charged (lysine, arginine, and histidine) and negatively-charged amino acids (aspartic acid and glutamic acid) were determined. Hydrophobic, hydrophilic, and aromatic amino acids were also identified. Locations of the transmembrane, intracellular, and extracellular regions were predicted using the TMHMM data bank (http://www.cbs.dtu.dk/services/TMHMM/). Post-translational modifications were predicted using the Center for Biological Sequence Analysis website at http://www.cbs.dtu.dk/researchgroups/PTM.php. The 3D structures of *PtrACS*1 and *PtrACS*2 were obtained from the SWISS-MODEL website at http://swissmodel.expasy.org/interactive.

### Phylogenetic tree and alignment

Sequences of the two ACS proteins and 19 protein sequences from other species were aligned with the CLUSTAL W program assembled using Mega 6.0 software (Tamura et al. 2011). A phylogenetic tree was drawn with Mega 6.0 using the neighbor-joining tree method with the p-distance substitution model, which was estimated by Mega 6.0 as the best-fit model. Reliability of the internal branches was assessed with 1000 bootstrap replicates and the values were marked above the nodes.

### Heterologous expression and purification of recombinant enzymes

The plasmid vector pET30a(+) (Qiagen, Hilden, Germany), digested by the *Kpn*I and *Sal*I restriction enzymes, was used to produce the recombinant proteins. The entire open reading frame, including the designed restriction enzyme sites, or the products obtained by digesting PMD18T-*ACS1* or PMD18T-*ACS2* with the respective enzymes, were inserted into pET30a(+) by ligation to form pET30a(+)-ACS1 or pET30a(+)-ACS2. These recombinant plasmids were transformed into *E. coli* BL21 cells and positive colonies were confirmed by sequencing. An overnight culture of *E.coli* BL21 transformed with recombinant plasmid was diluted 1:100 and grown in LB liquid media containing kanamycin (100 mg/L) until the absorbance at 600 nm (A600) reached 0.4–0.6. Isopropyl β-D-1-thiogalactopyranoside (IPTG) was added as the inducing agent at a final concentration of 0.3 mM, and incubated at 28 °C for 3 h. Cells were collected by centrifugation at 4000×*g* and 4 °C for 10 min and then resuspended in lysis buffer (50 mM NaH_2_PO_4_, 300 mM NaCl with 10 mM imidazole, pH 8.0). Cells were disrupted by sonication on ice for 150 cycles of 3-s pulses of maximal power and 7 s cooling between pulses, and the extracts were cleared by centrifugation at 12,000×*g* for 30 min at 4 °C. The protein was purified according to the manufacturer’s instructions for high-level expression and purification provided by QIAGEN, which was specific for the purification of 6 × His-tagged proteins from *E. coli* under native conditions. The purified target protein was kept at 4 °C. The sample (10 μg) and protein marker (5 μg) were loaded on a 12 % SDS-PAGE gel to verify the protein expression and purification results.

### Enzyme assay

4CL activity was determined by spectrophotometric assay (Knobloch and Hahlbrock 1977). The standard reaction mixture contained 2 μg of purified protein, 0.33 mM coenzyme A, 5 mM ATP, 25 mM MgCl_2_, and 500 mM Tris–HCl (pH 7.8). After incubation at room temperature for 10 min, the absorbance was monitored at wavelengths of 333, 363, and 345 nm for 4-coumaroyl-CoA, caffeoyl-CoA, and feruloyl-CoA, respectively. ACS activity was also determined by spectrophotometric assay. The reaction mixture contained 12.5 μL of 0.2 M MgCl_2_, 50 μL of 0.1 M ATP, 30 μL of 20 mM CoA, 30 μL of 0.2 M sodium acetate, and 50 μL of hydroxylamine solution. A volume of 450 μL of ferric-chloride reagent was adding to stop the reactions and the mixtures were kept on ice for 30 min. Next, the tubes were centrifuged for 2 min, and the red–purple color generated was measured at 540 nm with a UV-2102C spectrophotometer (Unico, Beijing, China). The extinction coefficient of acetyl hydroxamate was 0.975 mM^−1^ cm^−1^ under these conditions. Sodium acetate was used as the substrate for determining both pH and temperature optima. Phosphate buffer (10 mM) with a pH ranging from 5.0 to 9.0 was used to provide various pH conditions. The optimum pH of each ACS was fixed when analyzing the temperature profile. Enzymatic reactions were initiated by the addition of enzymes. All the mixtures were incubated for 10 min at each temperature before the reaction was initiated. The protein activity was measured by the method described earlier at temperatures of 4, 25, 30, 35, 37, 40, 50, and 55 °C, respectively. K_m_ and V_max_ values were determined by Lineweaver–Burk kinetics plotting in Excel software and using sodium acetate at concentrations ranging from 0.01 to 2 mM. The equation of K_*cat*_ = V_*max*_/[E] was used to calculate turnover number (K_*cat*_), where [E] refers to the active enzyme concentration and V_*max*_ to the maximal velocity.

### Quantitative real-time PCR analysis and ACS enzyme activity in *P. trichocarpa*

Total RNA was isolated from one-year-old *P. trichocarpa,* and the plant materials were divided into four parts; root, leaf, phloem, and xylem. The stem was divided into two parts using a scalpel, one part included the epidermis and phloem, (hereafter called the phloem), and the residual part of the stem we termed the xylem (Rao et al. 2015). Total RNA was isolated using the TransZol UP Kit (TransGen Biotech Co., Ltd.) according to the manufacturer’s instructions. Total RNA (2 μg) was reverse-transcribed into cDNA using Superscript II Reverse Transcriptase (Invitrogen, Carlsbad, CA, USA) and oligo(dT) primers. Gene expression was analyzed by quantitative real-time PCR using cDNAs equivalent to 100 ng of the total RNA. Gene-specific primers were designed for the PCR amplification (Additional file 1: Table S5). Quantitative real-time PCR assays were performed using the Mx3000P real-time PCR detection system (Stratagene, San Diego, CA, USA) and the Brilliant R SYBR Green QPCR Master Mix (Stratagene). Gene expression data were normalized using the *P. trichocarpa* alpha-tubulin gene (*TUA1*, accession number: POPTR_0002s11250.1) as a reference. Cycling parameters were as follows: 94 °C for 10 min, 30 cycles of 95 °C for 30 s, 60 °C for 30 s, and 72 °C for 30 s, and 40 cycles of 95 °C for 1 min and 60 °C for 30 s. Three plants were tested and all were analyzed separately. Error bars represent the standard error (SE) of independent triplicate assays. Data were analyzed using the iQ5 software (Bio-Rad, Hercules, CA, USA), and differences in gene expression were calculated using the 2–ΔΔC analysis method (Liu et al. 2013). Total proteins were extracted from root, leaf, phloem, and xylem using the TCA-acetone method (Saravanan and Rose, 2004). Total protein activity was determined using sodium acetate as the substrate and the same method as used for the enzyme assay.

## Results

### Molecular cloning and characterization of *PtrACS1* and *PtrACS2*

**Fig. 1:**
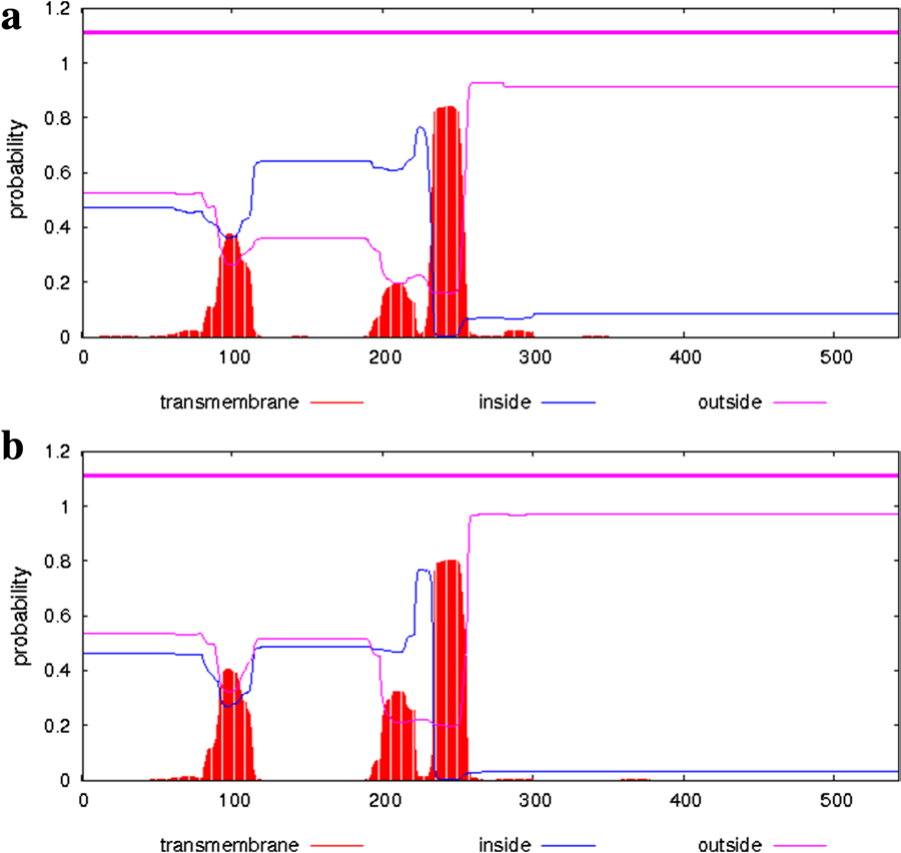
Transmembrane region prediction results for Ptr4CL6 (**a**) and Ptr4CL8 (**b**)

Based on the genomic sequence of the poplar genome (http://www.jgi.doe.gov/poplar/), we cloned and characterized two complete cDNA sequences from *P. trichocarpa*, Ptr4CL6 (accession number: XP_006373451) and Ptr4CL8 (accession number: XP_006373451). Primary structure prediction showed that the *Ptr4CL*6 and *Ptr4CL*8 cDNAs both contained a 1632-bp open reading frame (excluding the stop codon) and encoded peptides of 543 residues with predicted molecular masses of 59.21 and 59.43 kDa, respectively (Additional files 1: Tables S1 and S2). The isoelectric points (pI) of *Ptr4CL*6 and *Ptr4CL*8 were 8.84 and 8.74, respectively. The TMHMM program predicts the locations of transmembrane, intracellular, and extracellular domains. As seen in Fig. 1, Ptr4CL6 was predicted to have a positive transmembrane domain from valine 234 to leucine 255, while that of Ptr4CL8 from asparagine 233 to serine 253. Ptr4CL6 was predicted to have one uncertain transmembrane domain from proline 93 to alanine 104, while that of Ptr4CL8 from phenylalanine 96 to glicine 103 (Fig. 1). Post-translational modification results revealed that Ptr4CL6 and Ptr4CL8 were both modified post-translation. Phosphorylation, glycosylation and C-mannosylation sites were identified in both Ptr4CL6 and Ptr4CL8. No acetylation sites were identified in either of them (Additional file 1: Table S3).

**Fig. 2:**
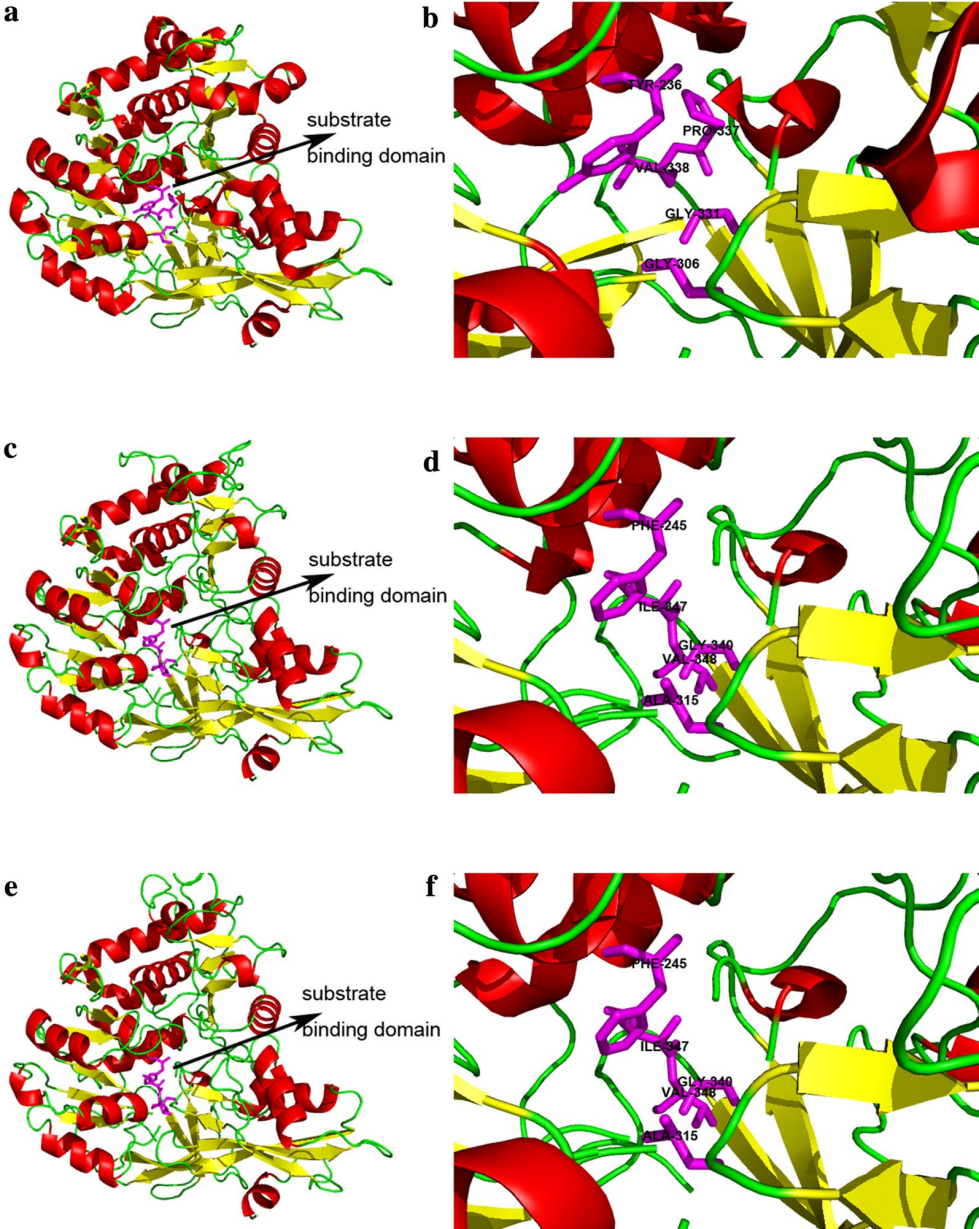
Three-dimensional structures and substrate binding domains of *Populus tomentosa* 4CL1 (**a**, **b**), Ptr4CL6 (**c**, **d**), and Ptr4CL8 (**e**, **f**)

The modeling and 3D structures were predicted using the SWISS-MODEL server, and the *Populus tomentosa* 4CL1 was most similar to Ptr4CL6 and Ptr4CL8, with similarities of only 42.39 and 42.86 %, respectively. Figure 2a, b shows the spatial model of *P. tomentosa* 4CL1, with its substrate binding sites Tyr-236, Gly-306, Gly-331, Pro-337, and Val-338 (Hu et al. 2010). Figure 2 shows the spatial model of Ptr4CL6 (Fig. 2c, d) and Ptr4CL8 (Fig. 2e, f), in which the substrate binding residues were changed on both to Phe-245, Ala-315, Gly-340, ILE-347, and Val-348. The change of the substrate binding residues may lead to Ptr4CL6 and Ptr4CL8 having a different catalytic activity than that of *P. tomentosa* 4CL1.

**Fig. 3:**
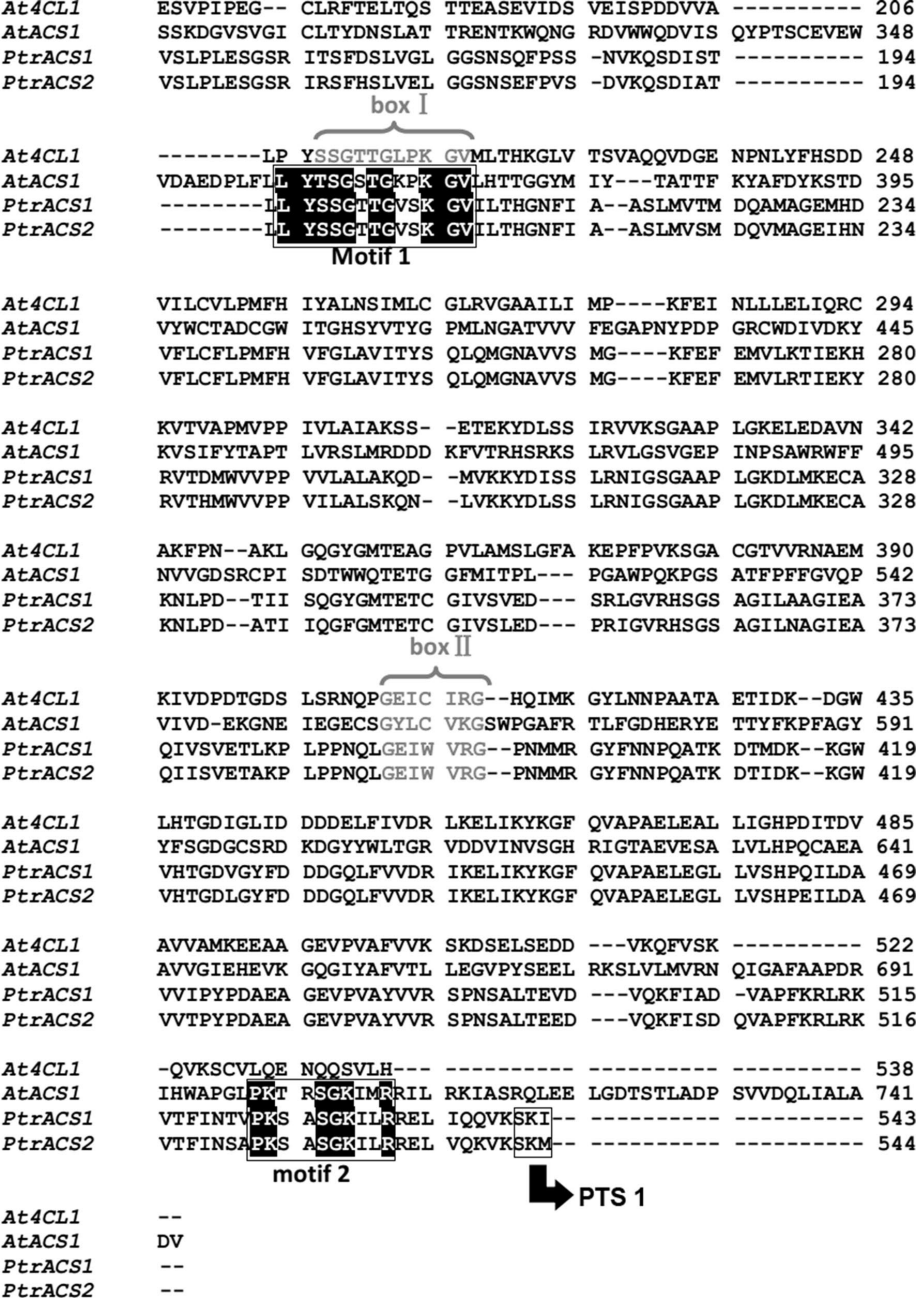
Amino acid sequence analysis of PtrACS1 and PtrACS2

Amino acid sequence analysis showed high similarity (92 %) between Ptr4CL6 and Ptr4CL8. NCBI BLAST analysis showed Ptr4CL6 and Ptr4CL8 to have 30–40 % sequence similarity to Ptr4CL. Sequence alignment revealed that the box I and box II domains (Martínez-Blanco et al. 1990) were conserved in Arabidopsis At4CL (Fig. 3), but were not conserved in Ptr4CL6 and Ptr4CL8. In particular, the main differences were a proline change to serine or valine in box I, and the combination of cysteine–isoleucine was substituted by tryptophan–valine. Box I was relatively conserved in the adenylate-forming enzymes superfamily, while box II was only conserved in 4CL. Based on the differences in box II between 4CL and our two 4CL-like genes, we speculated that Ptr4CL6 and Ptr4CL8 may not have the same catalytic activity as 4CL (Ehlting et al. 2005; Costa et al. 2005). However, compared with the AtACS1 protein, the conserved structure domains of motifs 1 and 2 in ACS were relatively conserved in Ptr4CL6 and Ptr4CL8 (Costa et al. 2005), which may suggest that Ptr4CL6 and Ptr4CL8 have ACS activity. The putative peroxisomal targeting signal type 1 (PTS1) in the C-terminal region, were the peroxisomal matrix targets sequences, displays an auxiliary targeting function (Wang et al.^1999^; Hayashi et al. 1997; Mullen et al. 1997; Kragler et al. 1998; Kato et al. 1998). Ptr4CL6 and Ptr4CL8 contained PTS1, indicating that both these Ptr4CL proteins may be located in the peroxisome and be related to lipid synthesis and metabolism. Based on this possibility, the *Ptr4CL*6 and *Ptr4CL*8 genes were renamed *PtrACS*1 and *PtrACS*2, respectively.

### Phylogenetic tree analysis

**Fig. 4:**
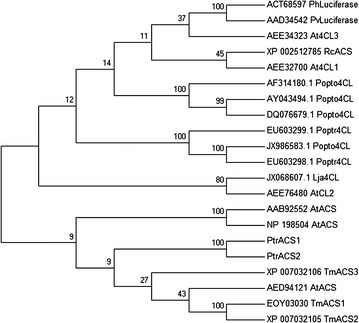
Phylogenetic tree showing the relationships between two PtrACS isoforms and other adenylate-forming superfamily members

To investigate the evolutionary relationships of the two genes with other adenylate-forming enzymes, we performed a phylogenetic analysis using the MEGA 6.0 program. The results clearly showed that PtrACS1 and PtrACS2 had high homology (Fig. 4, Additional file 1: Table S1), and had the closest evolutionary relationships with other ACS sequences (AAB92552 *Arabidopsis thaliana* ACS, NP_198504 *Arabidopsis thaliana* ACS, AED94121 *Arabidopsis thaliana* ACS, EOY03030 *Theobro macacao* ACS isoform 1, XP_007032105 *Theobro macacao* ACS isoform 2, and XP_007032106 *Theobro macacao* ACS isoform 3).

### Expression and purification of recombinant PtrACS1 and PtrACS2

The recombinant pET30a(+)/*PtrACS*1 and pET30a(+)/*PtrACS*2 vectors were transformed into the *E*.c*oli* strain BL21 to obtain recombinant proteins.

**Fig. 5:**
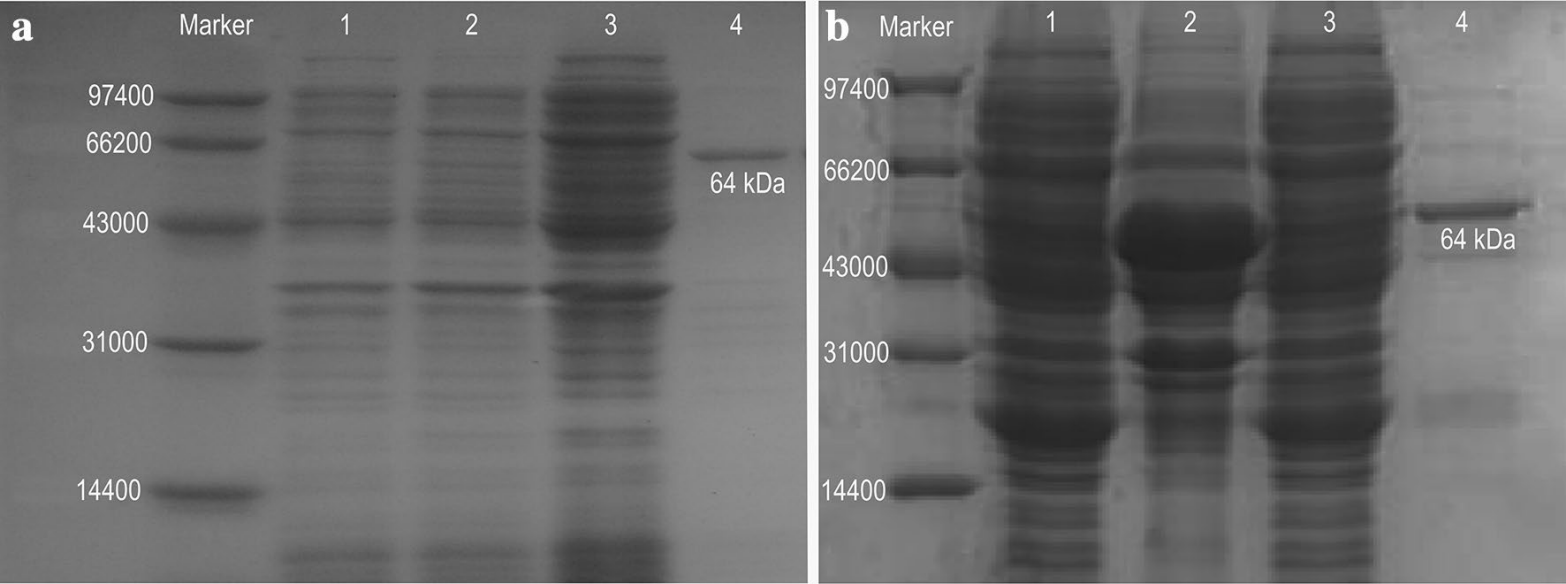
Expression and purification of recombinant PtrACS1 and PtrACS2. **a** Marker: Shanghai biochemical low molecular weight protein MARKER standard; *lanes 1* and *2*: total protein from BL21 (DE3) cells harboring pET-30a(+)-ACS1 not induced by IPTG; *lane 3*: total protein from BL21 (DE3) cells harboring pET-30a(+)-ACS1 induced by IPTG for 3 h; *lane 4*: 250 mM imidazole-eluted sample. **b** Marker: Shanghai biochemical low molecular weight protein MARKER standard; *lane 1*: total protein from BL21 (DE3) cells harboring pET-30a(+)-ACS2 not induced by IPTG; *lane 2*: total protein from BL21 (DE3) cells harboring pET-30a(+)-ACS2 induced by IPTG for 3 h; *lane 3*: the flow-through sample; *lane 4*: 250 mM imidazole-eluted sample

After inducing with IPTG for 3 h, the target proteins encoded by the *PtrACS1* and *PtrACS2* genes were expressed and then purified using the Ni–NTA column method under native conditions (Fig. 5a, b).

### Enzyme activity and kinetic analysis of recombinant PtrACS1 and PtrACS2

To further clarify the enzymatic characteristics of the recombinant PtrACS1 and PtrACS2, we examined their substrate specificities. In plants, 4CLs usually display high activity toward 4-coumarate, ferulate, and caffeate, while ACSs usually display high activity toward sodium acetate (Lindermayr et al. 2002; Hu et al. 1998; Ehlting et al. 1999; Hamberger and Hahlbrock 2004). We used various substrates (sodium acetate, coumarate, caffeate, and ferulate) to detect the activities of the recombinant PtrACS1 and PtrACS2. PtrACS1 and PtrACS2 showed high activity toward sodium acetate but little or no activity toward various cinnamic acid derivatives (coumarate, caffeate, and ferulate), with relative activities of PtrACS1 and PtrACS2 of 194.16 ± 11.23 and 422.25 ± 21.69 μM min^−1^ mg^−1^, respectively. This result provided additional proof that PtrACS1 and PtrACS2 belonged to the ACS protein family, and not to the 4CL protein family.

The steady state kinetics of both PtrACS enzymes were investigated in assays using 0.01, 0.02, 0.1, 0.2, 1.0, and 2.0 M sodium acetate. The kinetic parameters of the recombinant PtrACS1 and PtrACS2 were analyzed using a Lineweaver–Burk plot. The *K*_m_ and *V*_max_ for PtrACS1 were 0.25 mM and 698.85 μM min^−1^ mg^−1^, respectively, while for PtrACS2 they were 0.72 mM and 245.96 μM min^−1^ mg^−1^, respectively (Table 1, Additional file 2: Table S6a; Additional file 3: Table S6b).

**Table 1:** Enzymatic properties of PtrACS1 and PtrACS2 overexpressed in *E*. *coli*

	PtrACS1	PtrACS2
*K*_m_ (mM)	0.25 ± 0.02	0.72 ± 0.03
*V*_max_ (μM min^−1^ mg^−1^)	698.85 ± 32.05	245.96 ± 33.52
*k*_cat_ (min^−1^)	166.77	143.45
*k*_cat_/*K*_m_ (μM^−1^ min^−1^)	667.08	199.24
Optimal pH	7.5	8.0
Optimal temperature (°C)	35	35

### Effects of pH and temperature on enzyme activity and stability of recombinant PtrACS1 and PtrACS2

**Fig. 6:**
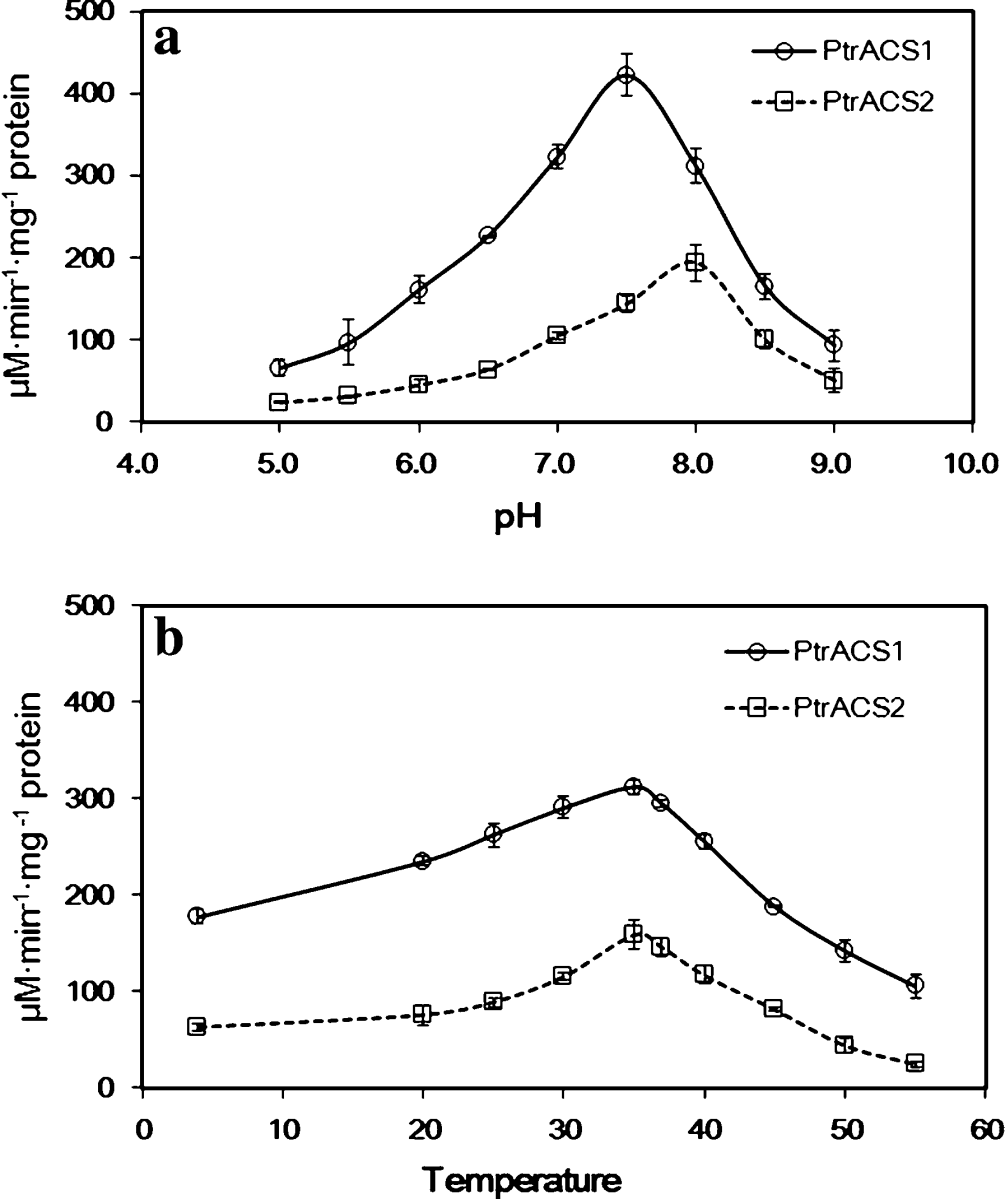
**a** pH profiles for recombinant PtrACS1 and PtrACS2; **b** Temperature profiles for recombinant PtrACS1 and PtrACS2

We evaluated the effects of pH on enzyme activity. The results showed that the activities of the recombinant PtrACS1 and PtrACS2 were pH-dependent. Recombinant PtrACS1 showed high levels of activity in a pH range of 7.0–8.0, with a pH optimum of 7.5. PtrACS2 functioned at a pH optimum of 8.0, and showed high levels of activity in a pH range of 7.5–8.5. Little activity was detected for either PtrACS1 or PtrACS2 at pH levels below 5.0 or above 9.0 (Fig. 6a).

Temperature profile analysis of the recombinant PtrACS1 and PtrACS2 indicated a temperature optimum for enzymatic activity of 35 °C. The enzymatic activities were >90 % of maximum over a broad temperature range between 25 and 40 °C, and both recombinant proteins retained >60 % of their maximum enzymatic activity at temperatures between 15 and 45 °C. PtrACS1 enzyme activity decreased to 12 % of its maximum at 55 °C (Fig. 6b). The enzyme activity trend at different temperatures was similar to that of the ACS isoenzyme from spinach leaves (Zeiher and Randall 1991).

### Gene expression patterns in *P. trichocarpa*

**Fig. 7:**
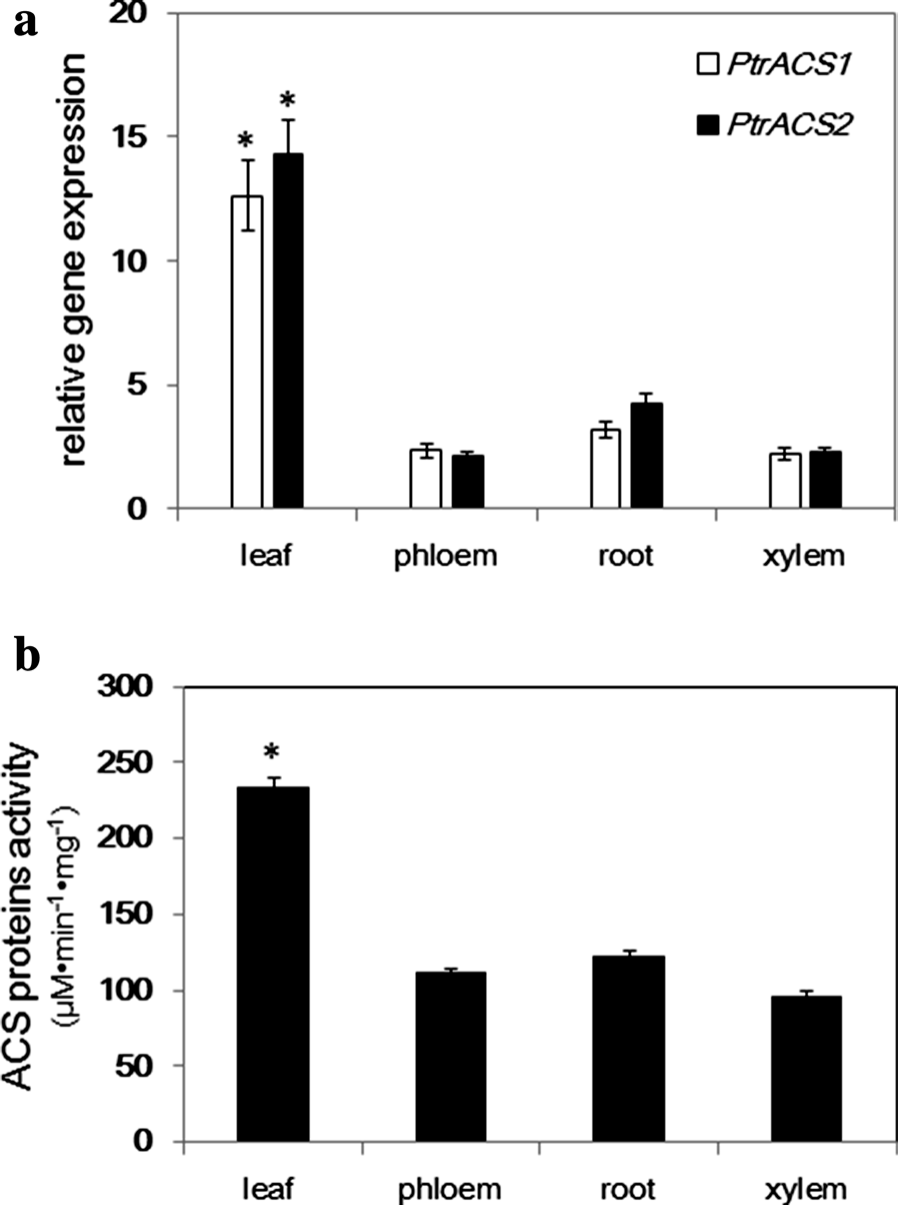
**a** Expression pattern of *PtrACS*1 and *PtrACS*2 in *P. trichocarpa*; **b** ACS protein activity in *P. trichocarpa*. Statistical comparison (Student’s *t* test) was made between leaf sample and other samples.*P < 0.05

To investigate the gene expression profile in various tissues, we measured *PtrACS*1 and *PtrACS*2 transcript levels quantitatively by quantitative real-time PCR in the xylem, phloem, leaf, and root of one-year-old *P. trichocarpa* using gene-specific primers. *PtrACS*1 and *PtrACS*2 were expressed in all of these tissues, indicating that their expression was widely distributed in the plant. However, *PtrACS*1 and *PtrACS*2 were expressed mainly in the leaves, while their expression levels exhibited no significant differences in the phloem, xylem, or root (Fig. 7a).

We detected ACS enzyme activity in various tissues of *P. trichocarpa* with all of the tissues tested showing activity toward the sodium acetate substrate. The highest activity was detected in the leaf, and lower activity was present in the root, phloem, and xylem; however, there were no significant differences between the phloem and xylem (Fig. 7b).

## Discussion

The adenylate-forming superfamily enzymes play a key role in lignin and flavonoid biosynthesis and, therefore, exploration of their biochemical characteristics has been a hot topic in plant metabolism research. However, the high level of structural similarities in the amino acids has made it difficult to identify single members, including ACS or the 4-coumarate:CoA ligases. 4CL activates 4-coumaric, caffeic, and ferulic acids to the corresponding CoA esters. These activated cinnamic acid derivatives serve as precursors for the biosynthesis of numerous plant secondary compounds, such as flavonoids, isoflavonoids, coumarins, lignin, suberin, anthocyanins, and wall-bound phenolics, which have essential functions in plant development and environmental interactions (Weisshaar and Jenkins 1998).

ACS (AMP forming; EC 6.2.1.1.) is an enzyme that first converts acetate and ATP into an acetyl-adenylate intermediate and then transfers the activated acetyl moiety to CoA, forming acetyl-CoA (Costa et al. 2005; Shockey et al. 2003; Reumann et al. 2004). Due to the high similarity in the protein structures of ACS and 4CL, it is difficult to determine the relationship between these two groups.

However, in vivo and in vitro enzyme activity analysis is a useful method to classify ACS and 4CL proteins into their respective groups. In this study, phylogenetic tree analysis revealed that some key residues, which were conserved among 4CL proteins, were changed in the PtrACS1 and PtrACS2 amino acid sequences, but PTS1 sequences, which represent a conserved domain of the ACS family, were found in the C-terminal regions of both PtrACS proteins. Although these two proteins displayed high similarity and identity to *P. tomentosa* 4CL1 based on 3D structural prediction, their similarity was quite low, and their substrate binding residues were different, which may result in different catalytic activity compared to 4CL. Taken together, this suggests that some key residues and targeting sequences, together with structural prediction should be helpful to distinguish the different classes of members in one family.

The recombinant PtrACS1 and PtrACS2 proteins did not react with the substrates of 4CL, but were able to react with the substrate sodium acetate, indicating that they belong to the ACS family. Compared to phylogenetic tree analysis and structure prediction, enzyme analysis could represent a better method to characterize these proteins.

PTS1 sequence has been founded at the end of the C-terminal of PtrACS1 and PtrACS2, suggests that they are targeted to peroxisome. The PTS1 sequence also reported in a group of acyl-CoA synthetase proteins from Arabidopsis, poplar and rice. Among of them, it was confirmed that PoptrACS5 subcellular localized in the peroxisome of mesophyll cell and epidermal cells using GFP fusion as a reporter (Souza Cde et al. 2008). In photosynthetic tissue ACS catalyzes the conversion of acetate to acetyl-CoA, which provides a key source for fatty acid, isoprenoid, and branched-chain amino acid biosynthesis (Kuhn et al. 1981; Zeiher and Randall 1991). This explains, to a certain extent, the higher gene expression levels of PtrACS1 and PtrACS2 in the leaves than in other tissues (Fig. 7a). The ACS enzyme activity results agreed with the gene expression profiles.

For many years, ACS was considered to be the primary source of acetyl-CoA for fatty acid biosynthesis, but metabolic and genetic studies have shown that this is not the case (Bao et al. 1998; Ke et al. 2000; Behal et al. 2002; Lin et al. 2003; Schwender et al. 2006). In research using Arabidopsis ACS mutants, ACS1 was shown to detoxify the products of aerobic fermentation (Lin and Oliver, 2008). Therefore, further studies are needed to understand how *PtrACS1* and *PtrACS2* regulate metabolism during *P. trichocarpa* growth, which metabolic process these ACS genes participate in, and the functional similarities and differences between them and other adenylate-forming enzymes.

## Conclusions

In this paper, we provided evidence from sequence analysis, and substrate specificity- and enzyme-activity assays to show that two 4CL-like genes, *PtrACS1* and *PtrACS2,* cloned from *P. trichocarpa* could be classified into the ACS family. Their high gene expression and ACS enzyme activity in leaves may suggest that they play a role in photosynthetic tissues.

## Additional files

10.1186/s40064-016-2532-7 **Table S1.** Physicochemical properties of PtrACS1. **Table S2.** Physicochemical properties of PtrACS2. **Table S3.** Predicted post-translational modification of PtrACS1 and PtrACS2. **Table S4.** The similarity between PtrACS1 and PtrACS2 as other Ptr4CLs. **Table S5.** Gene-specific primers used in real-time PCR analysis.
10.1186/s40064-016-2532-7 **Table S6a.** Enzyme activity and kinetic analysis of recombinant PtrACS1.
10.1186/s40064-016-2532-7 **Table S6b.** Enzyme activity and kinetic analysis of recombinant PtrACS2.
