# Supplementary material for: Enzymatic characterization of two acetyl-CoA synthetase genes from Populus trichocarpa
Source: Springerplus. 2016 Jun 21;5(1):818. doi: 10.1186/s40064-016-2532-7 (PMC4916118; doi:10.1186/s40064-016-2532-7)
Supplement: Supplementary file 1 — 10.1186/s40064-016-2532-7 Table S1. Physicochemical properties of PtrACS1. Table S2. Physicochemical properties of PtrACS2. Table S3. Predicted post-translational modification of PtrACS1 and PtrACS2. Table S4. The similarity between PtrACS1 and PtrACS2 as other Ptr4CLs. Table S5. Gene-specific primers used in real-time PCR analysis. [file 40064_2016_2532_MOESM1_ESM.docx]

Table S1. Physicochemical properties of PtrACS1

| Amino acid | No. of residues | % of residues |
| --- | --- | --- |
| Ala(A) | 35 | 6.4% |
| Arg(R) | 18 | 3.3% |
| Asn(N) | 21 | 3.9% |
| Asp(D) | 28 | 5.2% |
| Cys (C) | 4 | 0.7% |
| Gln (Q) | 19 | 3.5% |
| Glu (E) | 22 | 4.1% |
| Gly (G) | 41 | 7.6% |
| His (H) | 9 | 1.7% |
| Ile(I) | 37 | 6.8 |
| Leu (L) | 55 | 10.1% |
| Lys (K) | 38 | 7.0% |
| Met (M) | 16 | 2.9% |
| Phe (F) | 24 | 4.4% |
| Pro (P) | 32 | 5.9% |
| Ser (S) | 48 | 8.8% |
| Thr (T) | 27 | 5.0% |
| Trp (W) | 4 | 0.7% |
| Tyr (Y) | 14 | 2.6% |
| Val (V) | 51 | 9.4% |
| Pyl (O) | 0 | 0.0% |
| Sec (U) | 0 | 0.0% |

Table S2 Physicochemical properties of PtrACS2

| Amino acid | No. of residues | % of residues |
| --- | --- | --- |
| Ala(A) | 35 | 6.4% |
| Arg(R) | 20 | 3.7% |
| Asn(N) | 24 | 4.4% |
| Asp(D) | 24 | 4.4% |
| Cys (C) | 4 | 0.7% |
| Gln (Q) | 16 | 2.9% |
| Glu (E) | 27 | 5.0% |
| Gly (G) | 41 | 7.6% |
| His (H) | 10 | 1.8% |
| Ile(I) | 37 | 6.8 |
| Leu (L) | 57 | 10.5% |
| Lys (K) | 36 | 6.6% |
| Met (M) | 14 | 2.6% |
| Phe (F) | 26 | 4.8% |
| Pro (P) | 32 | 5.9% |
| Ser (S) | 49 | 9.0% |
| Thr (T) | 26 | 4.8% |
| Trp (W) | 4 | 0.7% |
| Tyr (Y) | 14 | 2.6% |
| Val (V) | 48 | 8.8% |
| Pyl (O) | 0 | 0.0% |
| Sec (U) | 0 | 0.0% |

Table S3. Predicted post-translational modification of PtrACS1 and PtrACS2

| Protein | Post-translational modification | Amino acid | Sites of residue |
| --- | --- | --- | --- |
| PtrACS1 | phosphorylation | serines | 56, 77, 119, 156, 161, 167, 184, 189, 198, 263, 307, 349, 360, 489, 525, 527, 541 |
|  |  | threonine | 166, 379, 495 |
|  |  | tyrosine | 6, 12, 113, 196, 402, 426 |
|  | O-glycosylation | serine | 156, 183, 541 |
|  |  | threonine | 102, 115, 517 |
|  | N-glycosylation | asparagine | 26 |
|  |  | serine | 28 |
|  |  | leucine | 27, 29 |
|  | C-mannosylation | tryptophan | 137, 285, 392, 418 |
|  | acetylation | --- | --- |
| PtrACS2 | phosphorylation | serine | 14, 28, 56 , 119, 161, 163, 167, 170, 177, 184, 189, 198, 263, 307, 349, 360, 489, 525, 527 |
|  |  | threonine | 282, 379, 412, 471, 495 |
|  |  | tyrosine | 6, 12, 113, 402, 426 |
|  | O-glycosylation | serine | 101, 163, 184, 197, 525, 541 |
|  |  | threonine | 115, 471, 517 |
|  | N-glycosylation | asparagine | 26 |
|  |  | serine | 28 |
|  |  | leucine | 27, 29 |
|  | C-mannosylation | tryptophan | 137, 285, 392, 418 |
|  | acetylation | --- | --- |

Table S4 The similarity between PtrACS1 and PtrACS2 as other Ptr4CLs

| Gene | Accession number | Similarity |
| --- | --- | --- |
| PtrACS1 | XP_006373451 | 100% |
| PtrACS2 | XP_006373451 | 91%ʹ |
| Ptr4CL | ACC63867 | 40.91% |
| Ptr4CL | XP_002297699 | 40.91% |
| Ptr4CL | XP_002304825 | 38.95% |
| Ptr4CL | ACC63868 | 38.95% |
| Ptr4CL | XP_002324477 | 37.94% |

Table S5 Gene-specific primers used in real-time PCR analysis

| Gene |  | Primer sequences |
| --- | --- | --- |
| PtrACS1 | Primer A | 5ʹ-CTGGTGCTGCCCCTCTGGG-3ʹ |
|  | Primer B | 5ʹ-GGAGGGAGAGGCTTTAGAG-3ʹ |
| PtrACS2 | Primer A | 5ʹ-CAGGCGCTGCTCCTCTGGG-3ʹ |
|  | Primer B | 5ʹ-CCACACTGATTATCTGGGC-3ʹ |
| TUA1 | Primer A | 5ʹ-CAGGACTGGAGCATACCGCCAGC-3ʹ |
|  | Primer B | 5ʹ-TGAAGGCCAGTGCAGTTATCAGC-3ʹ |
